# Editorial: The cognitive neuroscience of aging: where we are and where we are going

**DOI:** 10.3389/fnhum.2024.1476971

**Published:** 2024-08-28

**Authors:** Federico d'Oleire Uquillas, Ryan S. Falck, Barry S. Oken

**Affiliations:** ^1^Princeton Neuroscience Institute, Princeton University, Princeton, NJ, United States; ^2^School of Biomedical Engineering, University of British Columbia, Vancouver, BC, Canada; ^3^Vancouver Coastal Health Research Institute, Faculty of Medicine, University of British Columbia, Vancouver, BC, Canada; ^4^Rehabilitation and Technology (SMART) at Vancouver Coastal Health, Vancouver, BC, Canada; ^5^Departments of Neurology and Behavioral Neuroscience, Oregon Health & Science University, Portland, OR, United States

**Keywords:** cognitive function, aging, biomarkers and prevention, neurodiversity, cognitive enhancement

## Introduction

As our lifespan increases, so does our risk for cognitive decline and disorders associated with aging, such as Alzheimer's disease (AD) dementia. More than 50 million people were living with dementia in 2020, a number that could rise to 139 million by 2050, exceeding $1.3 trillion dollars in dementia-related costs.^1^ Given such societal and economic impact, and that the global population of adults older than 70 years old continues to outpace the growth of younger adults (Garmany et al., 2021), there is an increasing need for effective interventions targeting aging and cognitive impairment in these populations. This involves better understanding complementary markers of resilience across genetic, social, and biological constructs. To that end, here we review a set of recent findings that together provide an overview of the current state of the field and posit potential avenues for continuing to elucidate the cognitive neuroscience of aging.

## Social factors and cognitive health

Oken et al. highlight the critical need for interventions to address the insidious effects of loneliness and social isolation on cognitive health. Mid-life subjective feelings of loneliness have been shown to correlate with cognitive decline later in life (Akhter-Khan et al., 2021), dementia risk, and brain pathology (d'Oleire Uquillas et al., 2018), and its contribution to AD risk is even comparable to having a single APOE4 gene. Moreover, Oken et al. provide evidence that loneliness contributes to cognitive impairment independently of Alzheimer's pathology or other established risk factors. Their review underscores the importance of promoting social engagement as a strategy to maintain cognitive health in older adults.

## Cognitive reserve a protective factor

Factors that provide resilience against cognitive decline can be conceptualized as cognitive reserve (CR) (Stern et al., 2019). CR is pivotal in offsetting cognitive decline by allowing some individuals to maintain cognitive function despite age-related changes and brain injury (Stern et al., 2019). Borchers et al. have further explored this phenomena by investigating how the CR markers of IQ and educational attainment are related to postoperative neurocognitive disorders (PNDs). Their study demonstrates the role of CR in mitigating cognitive decline after surgery, but also notes the challenges in using CR markers, such as variability in preoperative cognitive status, and difficulties in standardizing anesthesia and postoperative treatment.

## Predicting cognitive decline

Curiel Cid et al. investigated psychometric predictors of progression in amnestic mild cognitive impairment (MCI) and identified proactive semantic interference (PSI) as an early detection marker of progression. Their findings suggest that individuals who exhibit higher levels of PSI may be at increased risk of developing dementia. By elucidating these predictive factors, Curiel Cid et al. contribute to a deeper understanding of the transition from MCI to dementia, and pave the way for more effective clinical interventions. Curiel Cid et al. also address limitations in their study on MCI progression, particularly pointing at the need for broader participant inclusion and longer follow-up periods. These findings align with broader themes addressed in the article collection “Longitudinal Aging Research: Cognition, Behavior and Neuroscience,” which highlights the importance of better understanding cognitive decline and aging trajectories: https://www.frontiersin.org/research-topics/11503/longitudinal-aging-research-cognition-behavior-and-neuroscience (Jäncke et al., 2022). Further, implementing routine cognitive testing during medical check-ups to establish baselines and detect early shifts in cognitive performance, before symptoms fully manifest, may significantly impact individual outcomes.

## Enhancing cognitive function through technology

In a usability study of neurotechnology for cognitive enhancement, Stramba-Badiale et al. demonstrated in MCI patients how technology can be harnessed to improve spatial navigation skills, a critical aspect of daily living often impaired in MCI and early dementia. By enhancing cognitive function through virtual reality (VR), they also raise important considerations regarding the accessibility and usability of VR technologies in clinical settings and beyond (e.g., insurance coverage, subsidies, etc.). Their findings emphasize the need for the development of policy that ensures equitable access to such emerging technologies. Overall, their findings suggest that user-friendly digital tools can be an effective aid in cognitive rehabilitation, and offer a promising target for non-pharmacological interventions in cognitive aging.

## Neurodiversity and aging

In their study of age-related differences in autistic individuals in the intrinsic connectivity of the hippocampus to the ventral lobe, circuits crucial for memory and spatial navigation, Chen et al. reveal that the aging process may look different across the neurodivergent spectrum. They call for inclusive research that considers diverse aging experiences in neurodiverse populations, and emphasize the importance of developing interventions for aging and cognitive decline that are tailored to meet the needs of neurodiverse individuals.

## Biomarkers in cognitive disorders

Understanding the neurobiological underpinnings of disease and age-related cognitive decline is essential for developing diagnostics and interventions. The review by Liu et al., discusses the role of neuron-specific enolase (NSE), a glycolytic enzyme found in neurons that is associated with neuronal damage and compromised cognitive function. NSE is emerging as a marker of brain health that may help facilitate the monitoring of cognitive decline and disease progression.

## Conclusion

**Figure 1:**
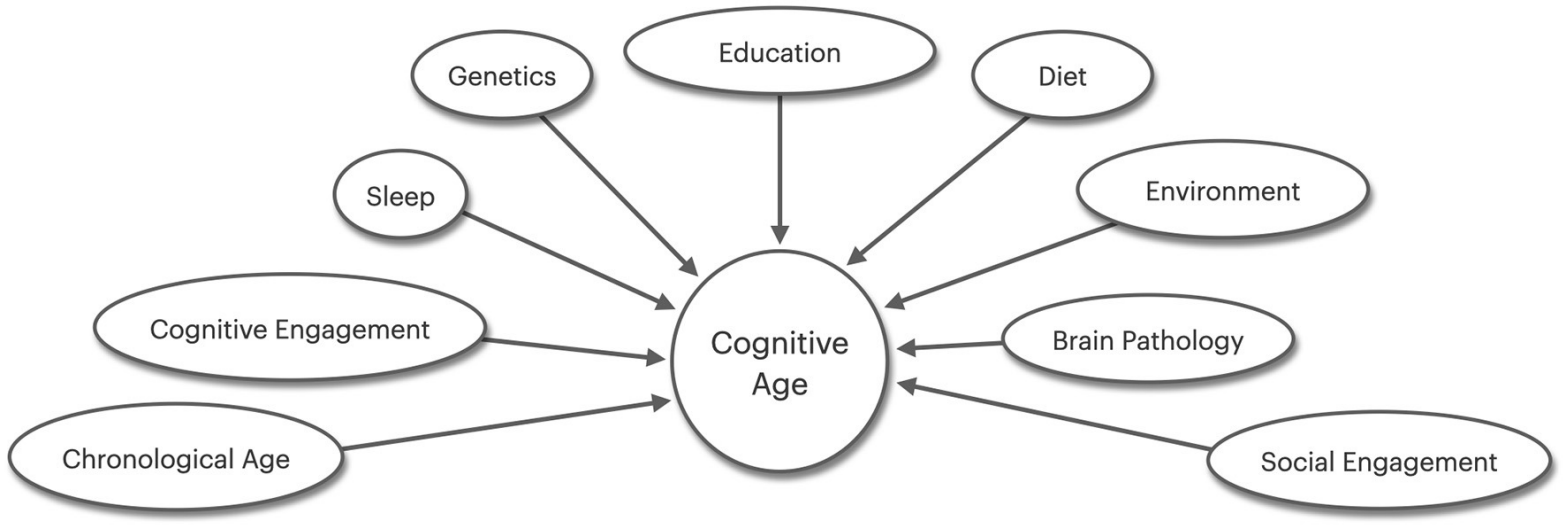
This figure highlights additional factors not directly discussed in the main text that may contribute to cognitive resilience and aging. A better understanding of how these may interact with established reserve mechanisms in supporting cognitive health in later life is crucial.

Promoting interdisciplinary collaborations and fostering equitable access to healthcare is essential for translating research findings into effective clinical practice and policy. Bridging gaps between neuroscience, psychology, public health, and geriatrics, also helps accelerate the development of therapeutic approaches for cognitive aging. In clinical settings, the integration of diverse reserve markers appears crucial, such as assessing social engagement, including the quality and quantity of social interactions. The figure above highlights other important reserve and aging factors not discussed here that may play a role in maintaining cognition in the face of increasing age, such as sleep, and dietary factors (Figure 1). As we gain a more nuanced understanding of cognitive aging, we ultimately improve the quality of life for aging populations.

## Footnotes

^1^Alzheimer's Disease International. Available at: https://www.alzint.org/about/dementia-facts-figures/dementia-statistics/.
